# An Ink-Jet Printed Eddy Current Position Sensor

**DOI:** 10.3390/s130405205

**Published:** 2013-04-18

**Authors:** Nikola Jeranče, Nikola Bednar, Goran Stojanović

**Affiliations:** Faculty of Technical Sciences, University of Novi Sad, Trg Dositeja ObradoviĆa 6, Novi Sad 21000, Serbia; E-Mails: bednar@uns.ac.rs (N.B.); sgoran@uns.ac.rs (G.S.)

**Keywords:** eddy current, position, printed, ink-jet, flexible inductor

## Abstract

An eddy current sensor with an ink-jet printed flexible inductor has been designed and fabricated. The inductor has been designed by means of software developed in-house. It has been fabricated by ink-jet printing with silver ink on a flexible substrate. The inductor is a part of the oscillator circuit whose oscillating frequency is measured by a microcontroller. The sensor characteristics have been analyzed for two types of application. The first considered application is the displacement of a large conductive target in a direction perpendicular to the inductor plane. The second considered application is the displacement of a small steel ball parallel to the inductor plane. Inductance and oscillating frequency have been measured in order to completely characterize the sensor. The obtained results validate the use of the sensor for both considered applications, and are in good agreement with the simulations. The advantages of this type of sensor are low cost, the possibility for the inductor to match any curved surface and flexibility and precision of the inductor design.

## 1. Introduction

Eddy current sensors are often used for the detection of conductive objects and for non-destructive testing. They allow the inspection of surface-breaking as well as subsurface discontinuities [1], fatigue cracks [2], material wear-out [3], profile imagery [4], *etc.* However, one of the most common applications of eddy current sensors is as position sensors [5]. In such a sensor, the change of impedance of a sensing coil in the presence of a conductive target is detected. Alternating current is fed to the sensing coil and its magnetic field induces eddy currents in the target which act on a sensing coil, reducing its inductance [6]. A coil-target distance measurement on a short stroke, up to one half of a coil diameter is possible [7]. The advantages of this type of sensor are: Absence of contact (*ergo* no wear-out), insensitivity to dirt and humidity, high temperature range, and no need for magnetic materials. A number of different technologies have been used to fabricate planar sensing elements for eddy current sensors, such as FPCB (flexible printed circuit board) [8], low temperature co-fired ceramics (LTCC) [9], on-chip microelectromechanical systems (MEMS) [10], or LC-LiGA [11].

Printed electronics, a rapidly emerging and relatively new technology within the electronics industry, is set to revolutionize the fabrication of electronic devices on flexible substrate materials such as plastic, paper, and textiles using electrically functional inks [12]. In recent years, there has been tremendous interest in the development of printed electronics components as a means of achieving ultra-low-cost and ubiquitous electronic circuits. Printed electronics using inkjet technologies have some advantages such as: direct printing of metal patterns on large and/or flexible substrates, the waste of coating materials is minimized [13], short design and fabrication time [14], mechanical flexibility [15] and wide range of new application [16,17]. Inkjet technology is a remarkable process whereby a small droplet of special liquid can be placed at a requested point with high accuracy and at a precise volume on flexible substrates such as poly(ethylene terephthalate) (PET), poly(ethylene naphthalate) (PEN) or polyimide (PI) [18,19].

In this paper, a new kind of sensing element for an eddy current sensor is studied: an inductor printed by ink-jet technology on a flexible substrate. The sensing element fabricated in this way could be embedded in curved surfaces, thus increasing the number of potential applications. Another advantage of this type of inductor is great accuracy and repeatability of the design. Ink-jet printing offers low-cost fabrication adapted to almost any particular application.

In [20] a flexible eddy current sensor is described, but it was fabricated using flexible printed circuit board technology, which has different technical parameters, such as higher conductor thickness and lower overall resistance of a printed coil.

Along with the usual application for this type of sensor—measuring the distance from a large conductive target, another use of the proposed sensor is studied—measurement of the distance from a small conductive target moving in a plane parallel (and close) to the plane of the printed inductor.

In the following section, an overview of different blocks of the sensor is given. In the Section 3, the design of the printed inductor is explained. In Section 4, the simulation method is explained and simulation results for the printed inductor in presence of conductive target are given. The practical realization of the sensor, including fabrication of sensing element, oscillator scheme and program for the microcontroller, is presented in Section 5. The measurement results obtained for the sensing element alone and for the whole sensor in practice are shown in Sections 6 and 7. Finally, conclusions with discussion of the results are given.

## 2. Overview of the Sensor

**Figure 1.**
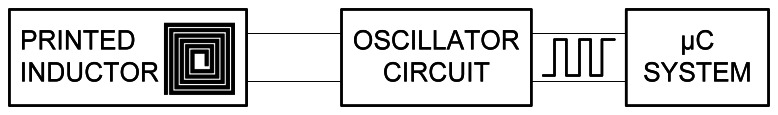
Sensor overview.

The sensor consists of three main parts (illustrated in Figure 1): Printed inductor, oscillator and microcontroller. The key part of the sensor is the printed inductor. Its inductance *L* changes in presence of a conductor. This variable inductance is a part of an electronic circuit whose frequency of oscillations will depend on the inductance *L*. The microcontroller is used for measuring the frequency of oscillations and displaying the results—oscillating frequency and presence/position of a conductive target.

## 3. Design of the Printed Inductor

**Figure 2.**
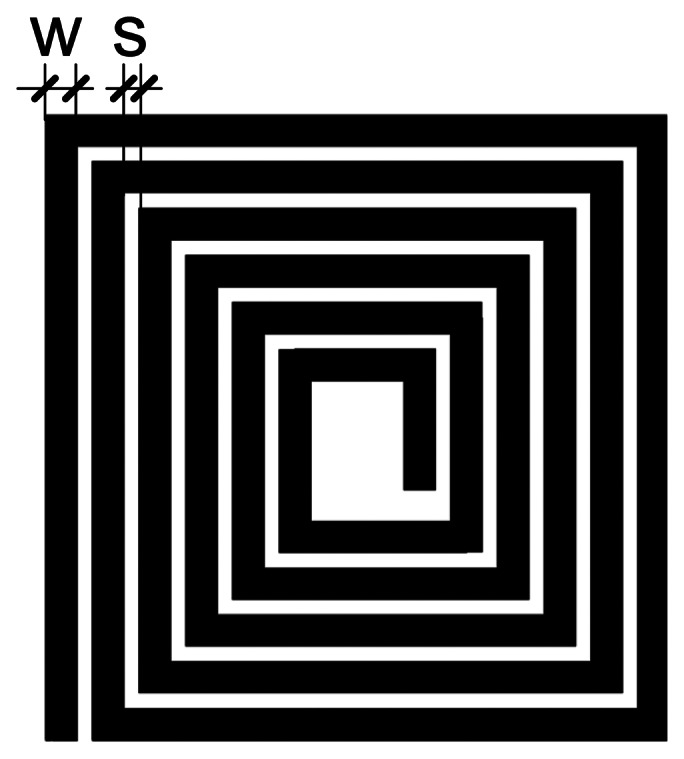
Spiral inductor.

The dimensions adopted for the design of printed inductor were 20 × 20 mm, in order to obtain a compact sensing device. The key requirement for the design is to achieve an inductance large enough for proper functioning of the oscillator and for reliable detection of the target. Too small an inductance can be difficult to detect in the circuit with other inductances present (wires, connections, *etc.*). Too high an inductance would decrease the oscillating frequency, thus decreasing the sensitivity. It should also be noted that high inductances are difficult to achieve in printed technology (they will also occupy a significant area). From previous experience with the oscillator, it was known that 800 nH inductance is sufficient to achieve these goals—in this case the oscillating frequency is close to 10 MHz. The resistance of such an inductor should also be taken into account, because of limitations of the printed conductors—the printed lines are very thin (less than 1 μm) and the resistivity of dried conductive ink is higher than the resistivity of the bulk material. From our measurements with passive components connected to the oscillator, it was known that the oscillator would function properly and in the convenient frequency range if the resistance is not much greater than 100 Ω. A spiral shape (Figure 2) of the sensor is convenient for maximizing the inductance.

Parameters which are used for the design are width of the conductor (*w*) and spacing between the adjacent conductors (*s*), as shown in Figure 2. These parameters can be changed within a reasonable range—too small a thickness would lead to a high resistance. Other limitations are due to the printing process itself–line widths or spacing smaller than a few hundred micrometers could lead to open circuits or short circuits between the adjacent conductors.

With these criteria in mind, the results of simulations, using the in-house developed software package Provod [21], are presented in Table 1. The expected resistance of the inductor is obtained from the average value of resistance per square for printed silver conductors determined in our laboratory. This value is 0.2 Ω per square.

The results in Table 1 show that, in order to maximize the inductance on a given surface, both *s* and *w* should be as small as possible. This is easily explained by the increased number of straight lines in the spiral, as the total surface is kept constant, and by increasing their self- and mutual inductances due to the smaller width and distance between them. Further decreasing of *s* and *w* would increase the resistance and would make the design more difficult to fabricate. The obtained inductance, for *s* = 0.5 mm and *w* = 0.5 mm, close to 900 nH, is sufficient and the expected resistance close to 90 Ω is not too high, therefore this design was adopted.

## 4. Simulation Results for the Printed Inductor in Presence of Conductive Target

**Figure 3.**
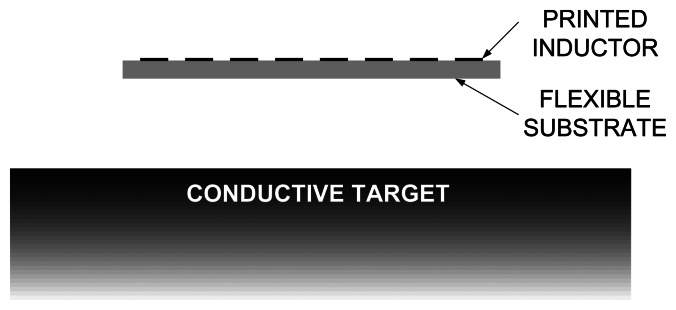
Cross section of the printed inductor in front of a conductive target.

In this section, the variation of inductance in proximity of a large flat conductive target is studied by computer simulations. The goal of these simulations is to estimate the change of inductance depending on the distance between the inductor and the target, shown in Figure 3. It should be mentioned that, if the distance from conductor to the target is needed, the substrate thickness must be added to the distance, because the printed inductor and the target are on the opposite sides of the substrate, as it can be seen in Figure 3.

The general equation of eddy current problem is as follows:

(1) $$\nabla^{2}A=\sigma \mu \frac{\partial A}{\partial t}$$

where *A* is magnetic vector potential, *σ* is the conductivity and *μ* is the permeability of the material.

**Figure 4.**
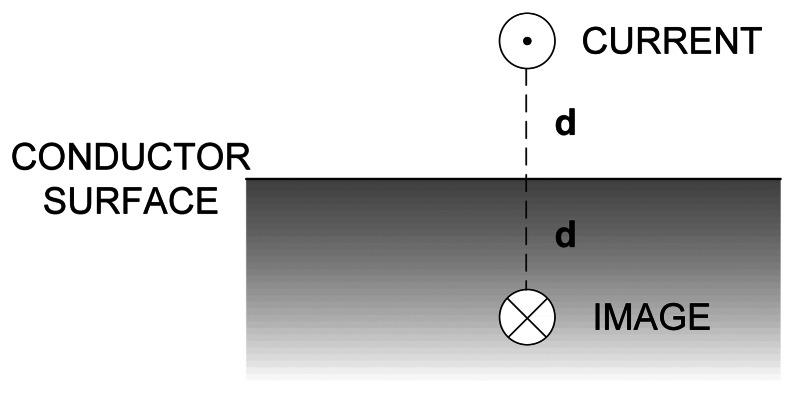
Method of images for eddy currents (approximation).

In a general case, its solution would require very demanding 3D finite element simulations. However, it is possible to simplify the problem by assuming that no magnetic flux penetrates into the material [22]. In this case, the magnetic potential vanishes at the boundary surface of the conductive material, therefore the conductive material can be replaced by negative image of the inductors' currents, as it is shown in Figure 4—each current in the system has its image current flowing in the opposite direction, at the same distance *d* from the conductors' surface. The Provod software package calculates inductances by integration through the current-carrying conductors [21]. The image of the printed inductor is easily entered and the simulation is done very quickly.

With this approximate method, we can simulate the inductor in order to give an estimate of the sensing range for the inductor adopted in Section 2. The quality of this approximation can be estimated by calculating the skin depth for a good conductor (for example Al) at the expected oscillator frequency.

The skin depth *δ* is defined as the thickness of the conductor is given by the following formula [22]:

(2) $$\delta =\sqrt{\frac{2}{\omega \sigma \mu }}$$

**Figure 5.**
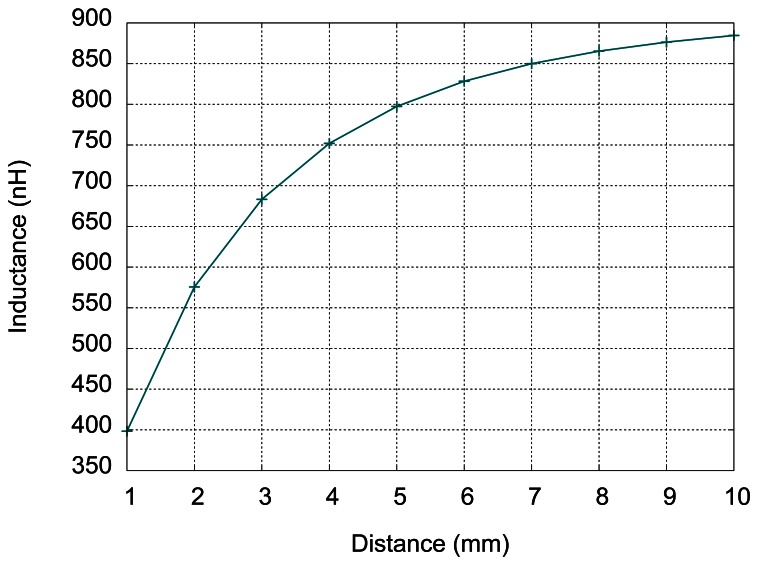
Simulation results: Inductance variation with distance inductor-target.

Knowing that the current density *J* decreases with depth *y* by exponential law [22]:

(3) $$J=J_{0}e^{\frac{-y}{\delta }}$$

where *J*_0_ is the current density at the surface, we can estimate the thickness of the layer with the currents as 3 to 5 times skin depth, which is a value close to 0.1 mm. Therefore, the simulation results are more accurate for larger distances (much greater than 0.1 mm) and less accurate for the target close to the inductor. The results for distances ranging from 1 to 10 mm (distance between the substrate and the target) are given in Figure 5.

According to the results presented in Figure 5, the sensitivity of the sensor is greater at small distances (less than 5 mm) and for larger distances, the inductance variations are more difficult to detect. At 10 mm distance, the inductance value for sensor without the target is almost reached. According to these simulations, the expected stroke of the sensor is between 6 and 9 mm, depending on desired sensitivity.

## 5. Practical Realization of the Sensor

### 5.1. Fabrication of the Inductor

**Figure 6.**
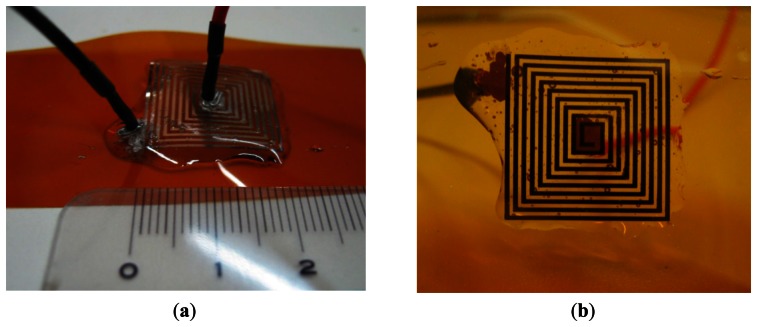
(**a**) Top and (**b**) bottom side of the printed inductor with contacts.

The designed inductor was fabricated using a Dimatix DMP-3000 (FUJIFILM Dimatix Inc., Santa Clara, CA, USA) materials deposition printer [23]. Commercial SunTronic Jet Silver U6503 silver ink was purchased from Sun Chemicals Ltd. (Slough, UK) and used to print conductive lines [24]. Drop spacing was set to 18 μm (resolution of loaded pattern image was 1,412 dpi), which results in optimum overlap between jetted droplets of ink (with a diameter of 50 μm, measured by using a camera image of the printer) in order to form conductive lines. Kapton film [25] with thickness of 75 μm was used as a substrate. After printing, the sensor was cured in an oven for 45 minutes at 200 °C. Wire contacts were connected with conductive silver epoxy paste, and dried for 10 minutes at 100 °C. In order to mechanically protect the sensitive printed layer and the contacts, they were sealed under a layer of dielectric epoxy, with 1.5 mm thickness. This step is also very useful to fix the sensor in a desired position after bending it around the desired surface. Top and bottom side of the fabricated inductor are shown in Figure 6.

Regarding the reproducibility of printed inductor, three identical structures were repeated on the same Kapton foil using ink-jet technology and two of them showed no fabrication errors, and had very close characteristics. One of them has been used for complete characterization of the sensor, which will be presented in Sections 6 and 7.

### 5.2. Oscillator Circuit

The printed inductor was employed as a part of an oscillating circuit, in a way that a change of its inductance affects the resonant frequency of oscillator. The oscillating (nearly sinusoidal) signal is passed through a Schmitt-triggering circuit and finally its frequency is measured using a microcontroller system.

There are many ways to construct an oscillating circuit with frequency that depends on the change of inductance of sensing element. One of the most commonly used types of oscillators is Colpitts oscillator, which comprises a resonant tank circuit formed by inductance *L* and the series combination of capacitances *C*_1_ and *C*_2_. Therefore, the operating frequency is approximately:

(4) $$f=\frac{1}{2\pi \sqrt{L\frac{C_{1}C_{2}}{C_{1}+C_{2}}}}$$

Using the presented formula, expected oscillation frequency of the circuit with employed printed inductor with inductance of 1,123 nH is 10.75 MHz.

**Figure 7.**
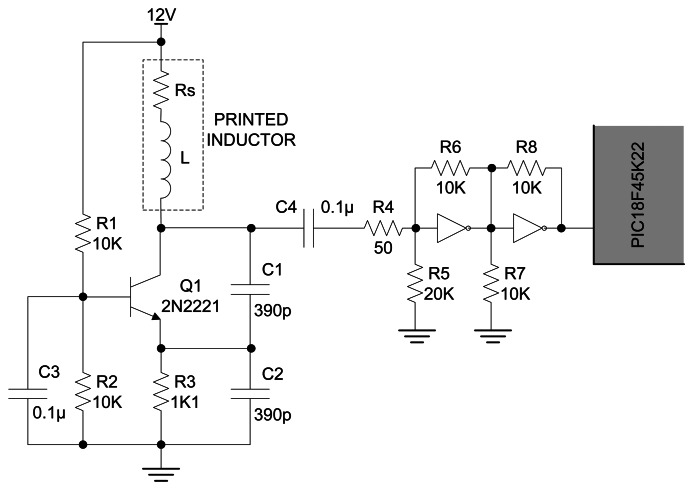
Complete electrical scheme of fabricated inductor as a part of oscillator circuit.

A Colpitts oscillator configuration with common-base has been adopted, due to its good response to unavoidable large serial resistivity of the printed inductor, which would prevent the oscillations in the tank circuit of some other oscillator configurations. The high load resistance of collector branch of the used oscillator even raises the amplitude of the output signal [20]. Voltage across *C*_2_ is applied to the base-emitter junction of the transistor *Q*_1_, as feedback to create oscillations. The transistor is properly polarized and kept in linear regime with resistors *R*_1_, *R*_2_ and *R*_3_. Complete scheme of used oscillator with voltage adjusting Schmitt-triggering circuit is presented in Figure 7.

### 5.3. Microcontroller Acquisition System

**Figure 8.**
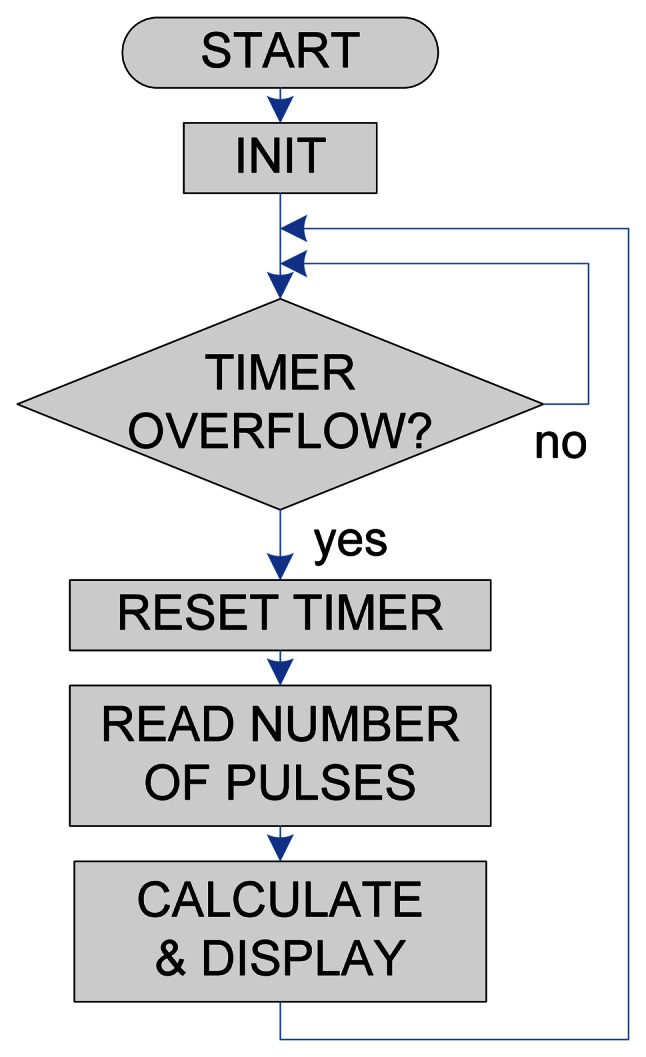
Program flow chart.

In order to measure the resonant frequency of the oscillator circuit and display the result a microcontroller based frequency meter was developed. Microchip's (Chandler, AZ, USA) PIC18F45K22 microcontroller was used for this purpose [26]. The frequency meter is based on counting of rising edges on the input pin (from the oscillator signal) in the fixed period of time, and calculating the frequency from this value. For this purpose two counter/timer modules were used. As the read frequency directly depends on the inductance of the used sensor, a look-up table with correlating data between these values was used. The software block diagram is presented in Figure 8. Reaction time of such a sensor system can be very fast. Reading frequency of the designed system was set to 100 μs, which is a good compromise between speed and accuracy of the measurement.

## 6. Measurement Results for the Printed Inductor

First measurements were done with inductor alone, in order to verify its self inductance, corresponding to the simulation result already presented in the Section 3. The impedance of the inductor with the contact wires has been measured using an HP4194A impedance analyzer. The impedance of the contact wires has been measured separately, in order to obtain the impedance of the inductor alone.

The following results have been obtained, at 10 MHz: total inductance of sensing element (printed inductor and contact wires) is 1,123 nH, the inductance of the wires alone is 240 nH. Therefore the inductance of the printed inductor alone is 883 nH, which is in a good agreement with the simulation result (917 nH) given in Table 1 in Section 3. The resistance of the inductor is 87.3 Ω, while the resistance of the wires is 0.2 Ω.

**Figure 9.**
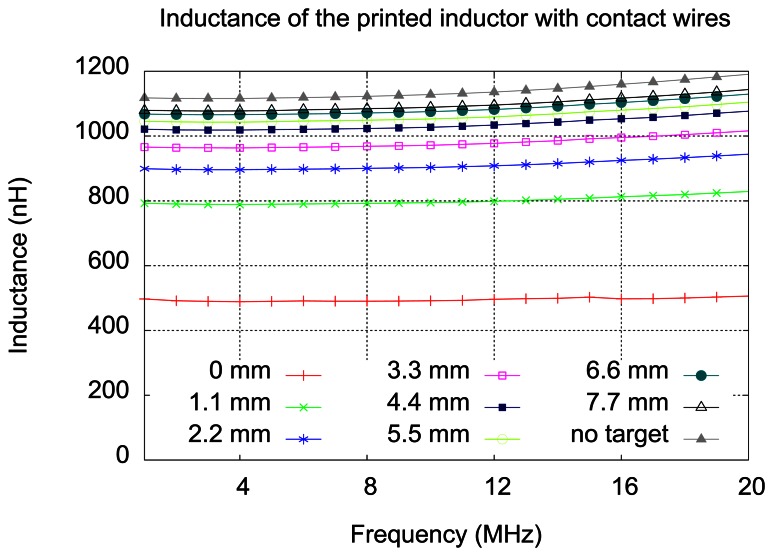
Measured inductance of the sensing element.

The following measurements were done for the inductor in front of a plate made of aluminum, corresponding to the simulations described in the Section 4. The distance between the inductor and the plate was created by inserting small flat pieces of glass. First set of measurements has been done with pieces of glass having 1.1 mm thickness. In Figure 9, measured inductance values for printed inductor with wires are shown, for the frequencies between 1 and 20 MHz, each curve representing one distance inductor-target. A curve for the inductor without the target is also added.

**Figure 10.**
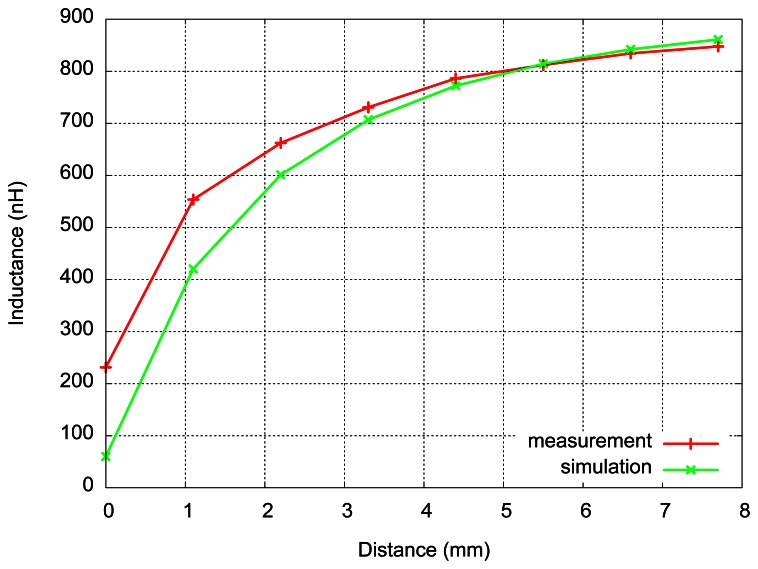
Inductance as function of a distance from conductive target: measured and simulated values.

We notice that the measured inductance increases with the distance from the target and it approaches the value of the inductance measured without the target. The inductance slightly increases with the frequency, due to the skin effect. The results of these measurements at 10 MHz, for printed inductor only, are given in Figure 10, together with the results of simulations made for 1.1 mm distance steps.

**Figure 11.**
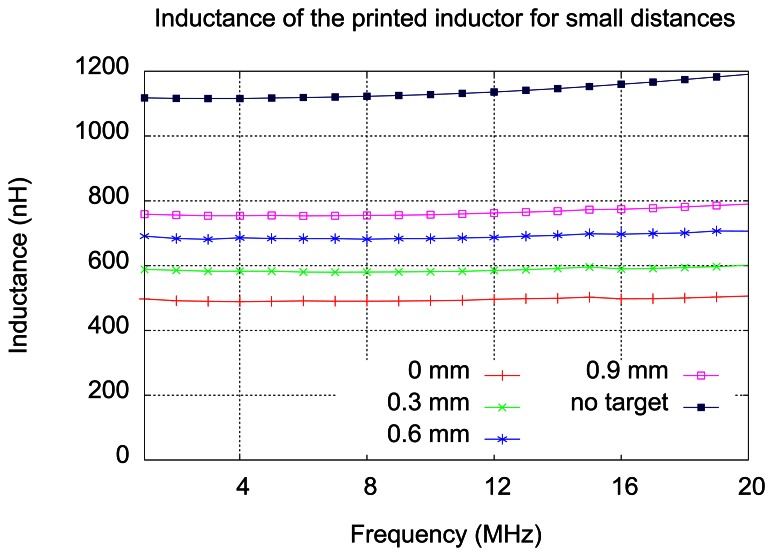
Inductance variation for small distances.

As expected, due to the approximation, the simulations are in good agreement with measurements for distances large enough (greater than 3 mm). The variation of inductance with distance becomes very small for distances larger than 5 mm. It can be noticed in Figure 10 that the slope of the curve decreases with distance. This consideration has lead to another measurement–this time the step distance was 0.3 mm, achieved by inserting thinner pieces of glass, in order to study the response of the sensor for small distances. The results of this measurement are presented in Figure 11.

For small distances (up to 1 mm) the inductor shows significant sensitivity—the inductance variation is close to 100 nH for 0.3 mm steps, at frequencies ranging from 1 to 20 MHz.

The second target was a small (8 mm diameter) steel ball from a bearing. The measurements with the second target are provided in order to illustrate the behavior of the sensor output in real world, with non-ideal targets. In this case, the measured decrease of inductance with the ball in the middle of the inductor's surface is 51 nH.

## 7. Measurement Results for the Sensor in Practice

**Figure 12.**
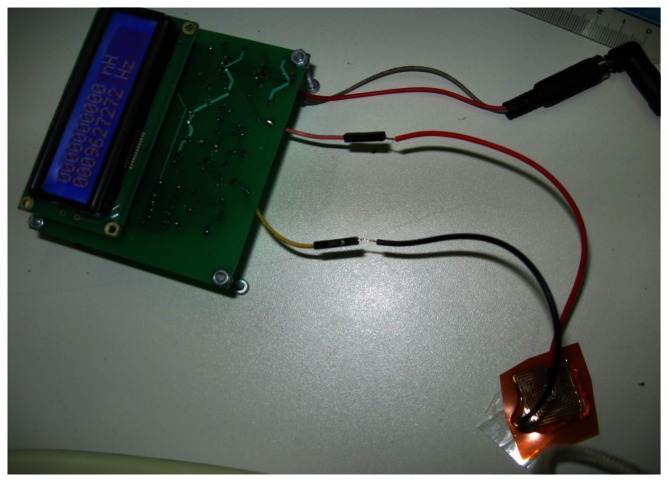
Complete sensor without target.

In Section 6, the behavior of the sensing element was validated with measurement of impedance. In this section, the testing of the whole sensor is described, including the oscillator and microcontroller for the two targets used for measurements presented in Section 6. The sensor without any conductive target in proximity is shown in Figure 12. Its oscillation frequency can be read on the display, and in this case it is 9.63 MHz.

**Figure 13.**
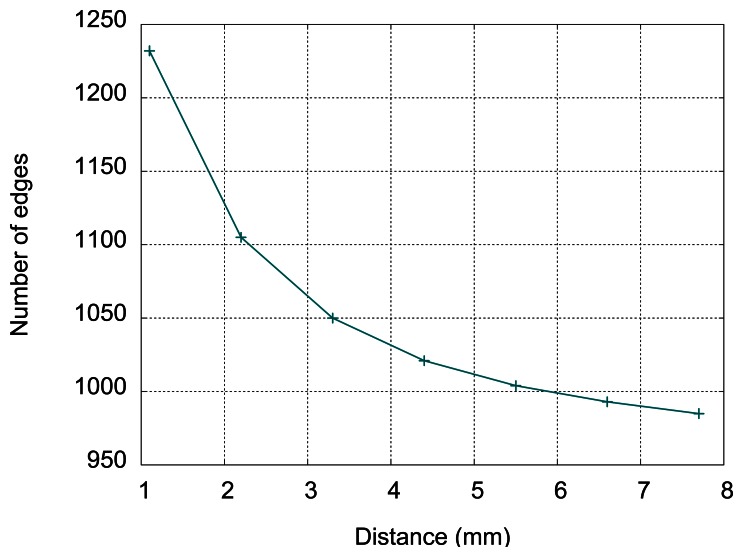
Measured number of edges with a large conductive target.

The measurements of oscillating frequency in respect to the distance between sensor and aluminum plate have been done, with 1.1 mm step, corresponding to Figure 10 in Section 6. The results (number of detected rising edges per 100 microseconds) are given in Table 2 and in Figure 13.

**Figure 14.**
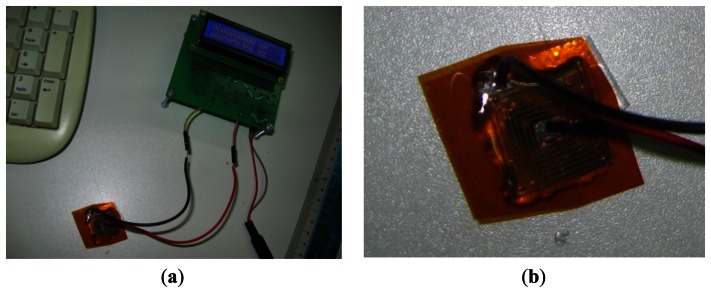
(**a**) Sensor detecting a metallic ball (**b**) Zoom: Printed inductor on top of the ball.

The sensitivity decreases with distance as can be seen from Figure 13. Up to 7.7 mm it is possible to distinguish consecutive target positions using the number of edges in 100 microseconds. For distances up to 7.7 mm, the presence of the target is easily detected. For distance close to 0, the number of edges is greater than 2,000, but the measurement becomes unstable (number of edges is variable and difficult to read on the display), due to low inductance in the oscillator circuit. This issue can be avoided by putting an additional inductance in series with the sensing element. In Figure 14 the sensor with the inductor placed on a metallic ball with 8 mm diameter is shown. The displayed frequency of oscillations is 9.86 MHz.

The difference in frequency is significant enough for detecting even a small target, such as a steel ball with 8 mm in diameter. For measurement time of 100 microseconds there is still enough pulses for accurate detection. One idea for an application is detecting the ball passing over the inductor in an arcade game.

**Figure 15.**
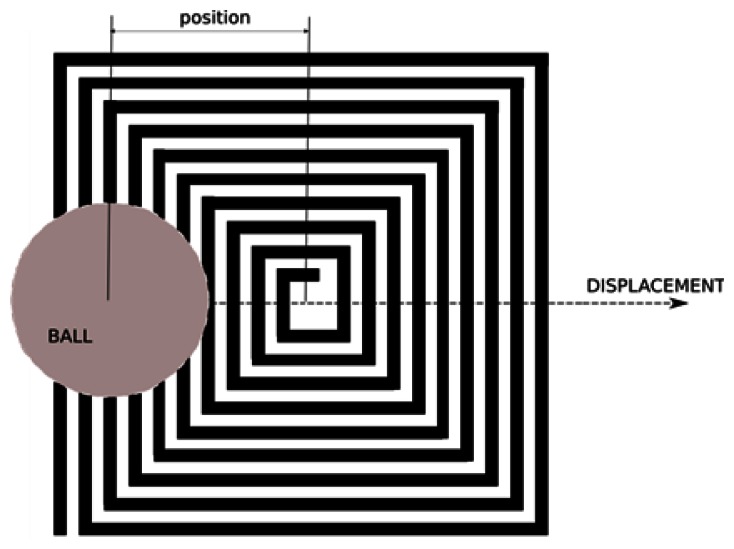
Drawing of the ball's position with respect to the center of the inductor.

For the displacement of the ball in the middle of the inductor, as it is shown in Figure 15, the output (number of edges counted during 100 microseconds) has been measured for several positions of the ball on the surface of the sensing element. In this case 967 edges were counted without the ball.

**Figure 16.**
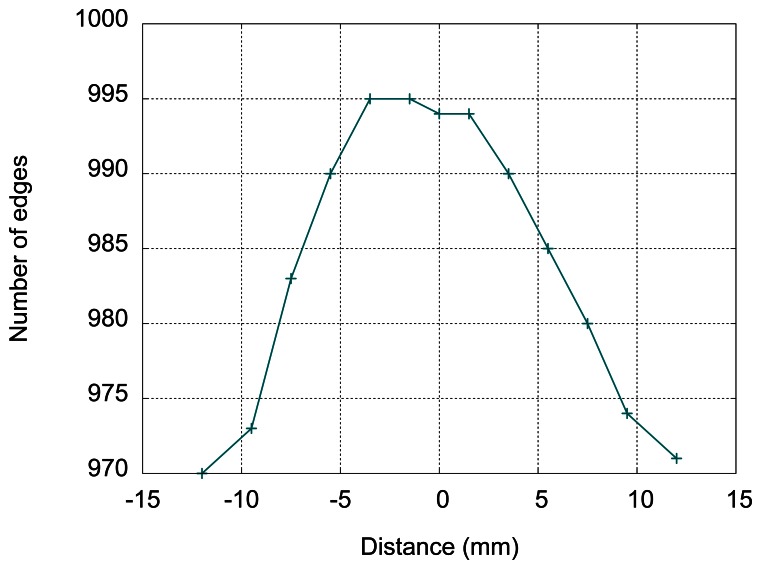
Measurement of sensor output for different positions of the steel ball.

The results are given in Figure 16, with the positions relative to the center of the inductor (please see Figure 15). The first and the last point are at 2 mm outside the inductor.

The number of detected edges varies with the position, and the threshold value can be adjusted to detect the presence of the ball in a smaller surface within the inductor, which suggests another use of this type of sensor for measuring the position of a small conductive target in a plane.

## 8. Conclusions

The sensing principle of an ink-jet printed eddy current position sensor has been thoroughly studied and validated, both using simulations and measurements. Furthermore, a complete sensor, including oscillator and microcontroller with a display has been fabricated and tested. A simple approximate simulation method is presented and compared with measurement results. Two types of position measurement have been studied: displacement of a large target perpendicular to the plane of the inductor and distance of a small target on the inductor's surface from the center of the inductor.

In the first case, the sensitivity for position measurement strongly depends on distance from the target. In the second case, even a small conductive target, such as steel ball of 8 mm in diameter (compared to the 20 × 20 mm inductor surface) can be reliably detected and its distance from the center of the sensing element can also be measured.

The design of the sensor is very dependent on the limitations of the fabrication, especially the printing of conductors. Therefore, the sensor described in this paper is likely to gain new applications, as printing technology progresses.

## Figures and Tables

**Table 1.** Simulation results for different inductor designs.

***s*(mm)**	***w*(mm)**	**Inductance (nH)**	**Expected resistance (Ω)**
0.5	2.0	147	16.6
1.0	1.0	259	42.9
0.5	1.0	404	54.3
0.5	0.5	917	86.1

**Table 2.** The number of detected rising edges as a function of the distance.

**Distance (mm)**	**Number of Detected Edges**
1.1	1,232
2.2	1,105
3.3	1,050
4.4	1,021
5.5	1,004
6.6	993
7.7	985
0	>2,000 (unstable)
no target	966
